# New Remedy for Scalds and Burns

**Published:** 1843-01

**Authors:** 


*New Remedy for Scalds and Burns.*—Mr. Wm. Rhind recommends as a remedy for burns and scalds, a solution of gum-arabic, repeated coats of it being applied, so as to form a complete covering to the injured parts. He relates several cases in which he tried it, and states that in all relief was procured in a very short time. The more recent the case, however, the more speedy was the removal of the pain. In those cases where blisters had appeared they were opened, and the solution applied; very frequently the application of the solution prevented the effusion of more serum; in some cases, however, serum was again effused and again evacuated.

In those distressing cases of the extensive burning of the bodies of young children, Mr. R. states that he would not hesitate applying the solution over the whole body, at about the warmth of 96°. It does not cool down the system (he remarks) by sudden evaporation or sudden abstraction of heat, like a common cold fluid, a circumstance in most cases to be dreaded, for gum is a bad conductor of heat; neither does it preclude an exposure to moderately cool air, which seems to keep down the excessive irritation consequent upon extensive scalding of the skin.

As it is of consequence to have the solution prepared instantly, the powdered gum, if it can be procured, may be in a few minutes dissolved in warm water. If this is not ready prepared, the common gum in small particles roughly pounded, will very soon dissolve, and the application in any case may be applied at a temperature of 90° or 100°, although in general it is more soothing when applied colder. Rancid gum solution should not be used, as it in this state has lost its adhesive quality. Two, three, or four applications may be necessary at intervals of five or ten minutes. The skin should be previously freed of all oily mattters, and the first coating, in order that it may be insinuated closely into the furrowed surfaces of the skin, should be rather thinner than the subsequent ones. In order to produce the proper effect it should form a varnished coat of some thickness and closeness over the whole space of the burnt part.—*Edinburgh Med. and Surg. Journ.* Oct., 1842.

